# Acadesine Circumvents Azacitidine Resistance in Myelodysplastic Syndrome and Acute Myeloid Leukemia

**DOI:** 10.3390/ijms21010164

**Published:** 2019-12-25

**Authors:** Thomas Cluzeau, Nathan Furstoss, Coline Savy, Wejdane El Manaa, Marwa Zerhouni, Lauriane Blot, Anne Calleja, Maeva Dufies, Alix Dubois, Clemence Ginet, Nicolas Mounier, Georges Garnier, Sophie Raynaud, Pierre Simon Rohrlich, Pierre Peterlin, Aspasia Stamatoullas, Fatiha Chermat, Pierre Fenaux, Arnaud Jacquel, Guillaume Robert, Patrick Auberger

**Affiliations:** 1 INSERM U1065, Université Côte d’Azur, C3M, 06204 Nice, France; nathan.furstoss@unice.fr (N.F.); coline.savy@unice.fr (C.S.); elmanaawejdane@live.com (W.E.M.); marwa.zerhouni@univ-cotedazur.fr (M.Z.); lauriane.blot@unice.fr (L.B.); calleja.a@chu-nice.fr (A.C.); maeva.dufies@gmail.com (M.D.); alix.dubois06@gmail.com (A.D.); clemence.ginet@unice.fr (C.G.); mounier.n@chu-nice.fr (N.M.); jacquel@unice.fr (A.J.); 2 Equipe Labellisée par la Ligue Nationale Contre le Cancer, 75013 Paris, France; 3 CHU de Nice, Service d’hématologie Clinique, 06200 Nice, France; sophie.raynaud@unice.fr (S.R.); rorhlich.ps@chu-nice.fr (P.S.R.); 4 Service de Médecine Interne, Centre Hospitalier Princesse Grace, Monaco 98000, Monaco; georges.garnier@chpg.mc; 5 Service D’hématologie Clinique, CHU de Nantes, 44093 Nantes, France; Pierre.peterlin@chu-nantes.fr; 6 Centre Henri Becquerel, 76038 Rouen, France; aspasia.stamatoullas@chb.unicancer.fr; 7 Service Hématologie Seniors, Hopital Saint-Louis, 75010 Paris, France; Fatiha.chermat-ext@aphp.fr (F.C.); pierre.fenaux@aphp.fr (P.F.)

**Keywords:** acadesine, azacitidine, apoptosis, MDS, AML, Phase I/II clinical trial

## Abstract

Myelodysplastic syndrome (MDS) defines a group of heterogeneous hematologic malignancies that often progresses to acute myeloid leukemia (AML). The leading treatment for high-risk MDS patients is azacitidine (Aza, Vidaza^®^), but a significant proportion of patients are refractory and all patients eventually relapse after an undefined time period. Therefore, new therapies for MDS are urgently needed. We present here evidence that acadesine (Aca, Acadra^®^), a nucleoside analog exerts potent anti-leukemic effects in both Aza-sensitive (OCI-M2S) and resistant (OCI-M2R) MDS/AML cell lines in vitro. Aca also exerts potent anti-leukemic effect on bone marrow cells from MDS/AML patients ex-vivo. The effect of Aca on MDS/AML cell line proliferation does not rely on apoptosis induction. It is also noteworthy that Aca is efficient to kill MDS cells in a co-culture model with human medullary stromal cell lines, that mimics better the interaction occurring in the bone marrow. These initial findings led us to initiate a phase I/II clinical trial using Acadra^®^ in 12 Aza refractory MDS/AML patients. Despite a very good response in one out 4 patients, we stopped this trial because the highest Aca dose (210 mg/kg) caused serious renal side effects in several patients. In conclusion, the side effects of high Aca doses preclude its use in patients with strong comorbidities.

## 1. Introduction

Myelodysplastic syndromes (MDS) represent a group of myeloid neoplasms characterized by blood cytopenia and an increased risk of leukemic transformation. Due to the progressive aging of the population in western countries, the number of MDS patients will significantly increase in the next decades. In this context, MDS will be one of the most challenging issues for hematologists and health care providers in the near future. Azacitidine (Aza, Vidaza^®^), a pyrimidine nucleoside analog is routinely used as the leading treatment for myelodysplastic syndromes (MDS) and acute myeloid leukemia (AML) patients ineligible for intensive chemotherapy [1,2]. In MDS patients treated with Aza the overall survival is significantly prolonged by 10 months versus conventional care. Unfortunately, 40% of patients are primary refractory to this drug and 60% of initial responders will relapse in an undefined period. After failure or relapse, overall survival (OS) of these patients is very poor [3].

Aza is a hypomethylating agent that induces apoptosis and cell cycle arrest [4]. We have previously characterized a defect in the mitochondrial pathway of apoptosis in Aza-resistant cells [5]. Concomitantly, we identified a high level of basal autophagy in Aza-resistant cell lines and proposed that induction of autophagic cell death could be a pertinent therapeutic strategy to eradicate Aza-resistant cells [6]. Acadesine (Aca, Acadra^®^) is another nucleoside analog that has proven anti-leukemic effect in hematopoietic malignancies. Indeed, it was shown to induce apoptosis in B-cell chronic lymphocytic leukemia [7,8,9,10] and also autophagic cell death in chronic myelogenous leukemia cells [11]. Aca is an AMP mimetic that can induce autophagic cell death via activation of AMPK and inhibition of the mTOR pathway or both [7,12,13,14].

Autophagy is an essential lysosomal catabolic pathway for the recycling of macromolecules, bulk cytoplasm and damages organelles. It exerts a paradoxical role in the control of cell death and survival [15]. Autophagy is activated under stress conditions such as nutrient deprivation, hypoxia and drugs treatment. The main function of autophagy is to refuel energy to the cell to promote cell survival under adverse conditions, but there is increasing evidence that autophagy can serve *per se* as a cell death mechanism under certain circumstances [16] and can also favor emergence of tumor initiating cells [17,18,19,20].

The aim of this study was to evaluate in vitro, in vivo and ex vivo the effect of Aca in MDS and AML cell lines and bone marrow cells from MDS/AML patients. We found that Aca was highly efficient to eliminate Aza-resistant cell lines and primary bone marrow myeloid cells from Aza-sensitive and resistant MDS and AML patients. A phase I/II clinical trial devoted to investigating the efficacy of Aca in Aza-refractory patients was initiated. However, due to serious renal side effects of Aca at the higher dose used in the study, the trial stopped and only 4 patients were fully analyzed. Among them, one exhibited a very good response (80% decrease in blast count). Although high doses of Aca in humans may have a side effect that precludes a chronic use in elderly patients, lower doses of Aca in combination with Aza might could be evaluated in MDS and AML patients.

## 2. Results

### 2.1. Aca Induces Cell Death in an Apoptosis-Independent Manner in MDS Cell Lines

**Figure 1:**
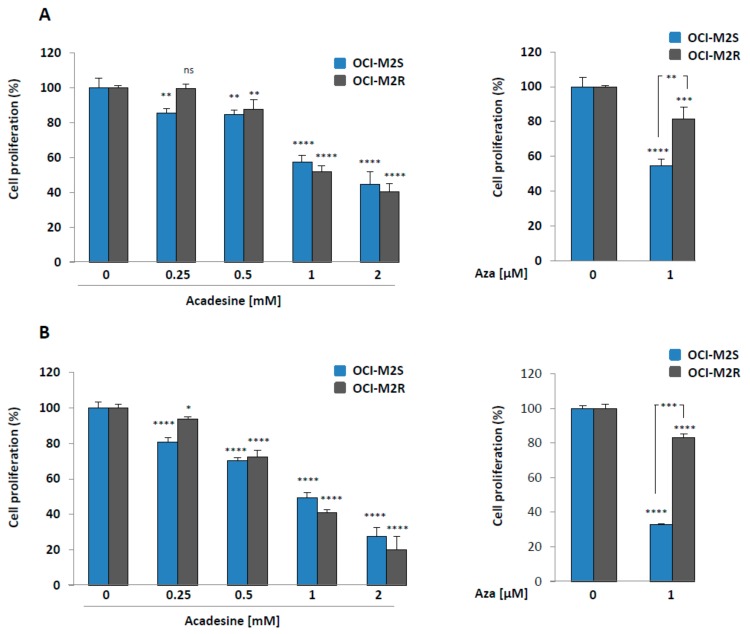

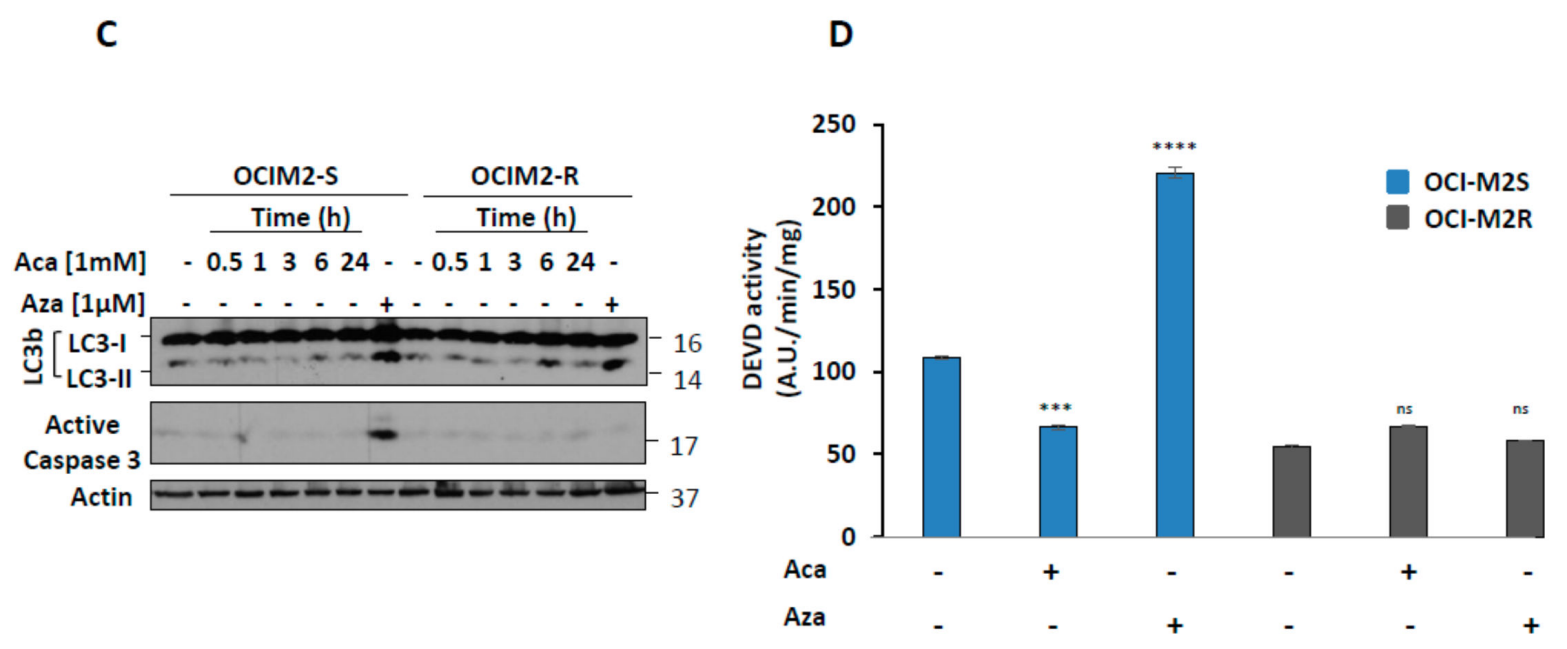
Aca exerts anti-leukemic effects on both OCI-M2S and OCI-M2R cell lines (**A**) Left panel, OCI-M2S and OCI-M2R cells (0.5 × 10^6^ cells/mL) were treated or not with increasing concentrations of Aca for 24 h at 37 °C. Right panel, OCI-M2S and OCI-M2R cells (0.5 × 10^6^ cells/mL) were treated or not with 1µM Aza, then in a both experiments cell metabolism was measured using the XTT assay as described in Materials and Methods section. Results are means ± SD of 3 different determinations made in triplicate. (**B**) Left and right panels, OCI-M2S and OCI-M2R cells (0.5 × 10^6^ cells/mL) were treated and analyzed as described in Figure 1A for 48 h at 37 °C. (**C**) OCI-M2S and OCI-M2R cells (1 × 10^6^ cells/mL) were incubated for various times at 37 °C with 1 µM Aza or 1 mM Aca. Whole-cell lysates were prepared, and expression of Poly-ADP-Ribose polymerase (PARP), LC3 and caspase 3 was visualized by western blotting. Actin was used as loading control. (**D**) OCI-M2S and OCI-M2R cells (0.5 × 10^6^ cells/mL) were treated or not with 1 mM Aca or 1 μM Aza for 24 h at 37 °C. Cells were harvested, washed, and lysed in caspase buffer. Caspase-3 activity was evaluated in quadruplicate using Ac-DEVD-AMC as substrate. To allow specific assessment of caspase activity, hydrolysis was followed as a function of time in the presence or the absence of 10 mM Ac-DEVD-CHO. Results were expressed as arbitrary units (a.u.) per min and per mg of proteins and are the means +/− SD of 4 independent experiments performed in quadruplicate. * *p* < 0.05, ** *p* < 0.01, *** *p* < 0.001, **** *p* < 0.0001, ns: not significant.

**Figure 2:**
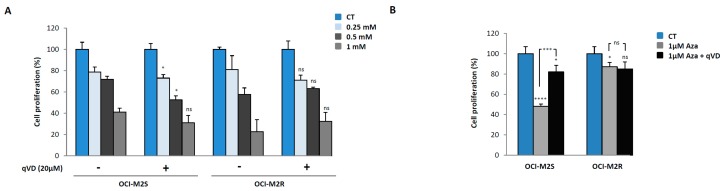
The anti-leukemic effects of Aca are mediated by caspase independent mechanisms (**A**) OCI-M2S and OCI-M2R cells (0.5 × 10^6^ cells/mL) were treated with increasing concentrations of Aca for 48 h at 37 °C, in the presence or in the absence of 20 µM Z-VAD-fmk, a pan-caspase inhibitor. Cell metabolism was measured using the XTT assay as described in Materials and Methods section. (**B**) OCI-M2S and OCI-M2R cells (0.5 × 10^6^ cells/mL) were treated with 1 µM of Aza for 48 h at 37°C, in the presence or in the absence of 20 µM Z-VAD-fmk, a pan-caspase inhibitor. Cell metabolism was measured using the XTT assay as described in Materials and Methods section. * *p* < 0.05, *** *p* < 0.001, **** *p* < 0.0001, ns: not significant.

Nucleoside analogs are mandatory molecules for the treatment of MDS and AML patients as exemplified by the beneficial therapeutic effect of Aza in both hematopoietic malignancies. Aca is also a nucleoside analog that has shown promising effect in vitro in B cell chronic lymphocytic leukemia [7,8], mantle cell lymphoma [21] and chronic myelogenous leukemia [11,22]. This prompted us to investigate the anti-proliferative and cell death inducing capacity of Aca in myeloid leukemia and more particularly in MDS/AML cell lines. As resistance to Aza is a consistent hallmark of MDS patients [6,23], we also took advantage in the present study of the availability of an Aza-resistant cell line (OCI-M2R) recently generated by our team. As expected, and conversely to Aza-sensitive OCI-M2S cells, OCI-M2R cells were resistant to this drug at 24 and 48 h (Figure 1A,B, right panels). Of note, Aca induced a dose-dependent loss of cell proliferation in OCI-M2S and OCI-M2R cells, as well. In both cell lines, a maximal inhibition of cell proliferation was obtained for 2 mM Aca (Figure 1A,B, left panels) and the dose of Aca triggering 50% inhibition of cell proliferation (IC_50_) was less than 1 mM at 48 h (Figure 1B, left panel), which is in the range of Aca effect’s in other hematopoietic cell lines. We next look for the effect of Aza and Aca on both apoptosis induction and LC3-II accumulation in OCI-M2S and OCI-M2R cells. Aza (1 μM) triggered caspase-3 cleavage at 24 h in OCI-M2S cells but not in their Aza-resistant counterpart, as expected (Figure 1C). At the same time, Aca failed to induce caspase 3 cleavage in OCI-M2S and OCI-M2R cells. Aza was also found to increase LC3-II accumulation in both cell lines, while Aca only moderately increased LC3-II conversion at 6 h in OCI-M2R cells (Figure 1C). Increased caspase-3 enzymatic activity was detected in OCI-M2S cells treated 24 h with Aza, but not in OCI-M2R cells (Figure 1D). Conversely to Aza, Aca failed to increase caspase 3 activity in sensitive and resistant lines. Rather, Aca slightly inhibited caspase 3 activity in OCI-M2S cells (Figure 1D). The increase in caspase 3 induced by Aza matched with the induction of apoptotic cell death already reported in other MDS/AML sensitive cell lines [6,23]. Finally, Aca failed to induce caspase activation in sensitive and resistant cell lines as well, as anticipated from the lack of caspase 3 cleavage illustrated in Figure 1C. As caspase activation was observed following Aza but not Aca treatment, we further investigated the role of apoptosis in the effect of both drugs. Thus, we first assessed the effect of qVD, a pan-caspase inhibitor on Aca-mediated loss of cell proliferation in OCI-M2S and OCI-M2R cells (Figure 2A). The effect of different concentrations of Aca on OCI-M2S and OCI-M2R cell proliferation was unaffected by qVD a pan-caspase inhibitor, indicating that it does not rely on apoptosis induction (Figure 2A), whereas the effect of Aza was impaired by qVD in OCI-M2S cells (Figure 2B) in agreement with the known proapoptotic effect of this drug. As expected, Aza failed to affect cell proliferation in OCI-M2R cells. These findings highlight the implication of another mode of cell death distinct from apoptosis and likely from autophagy in the mechanism of action of Aca.

### 2.2. Aca Efficiently Kills Primary Cell from Aza-Resistant MDS and AML Patient ex vivo.

**Figure 3:**
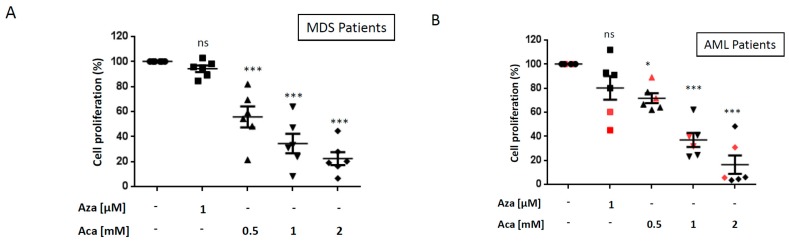
Aca exerts potent anti-leukemic effect in MDS and AML bone marrow cell ex vivo. Bone marrow cells from Aza refractory MDS/AML patients (10^6^/mL) were incubated for 24 h with 1 µM Aza or different concentration of Aca. Loss of cell proliferation was assessed using the XTT assay as described in Figure 1. Each assay was performed in quadruplicate. The mean values for each MDS patient and each AML patients are shown on Figure 3 (**A**) and (**B**) respectively. * *p* < 0.05, *** *p* < 0.001, ns: not significant. The bone marrow cells from two patients (in red) were slightly sensitive to Aza.

The effect of Aca was next investigated in 6 high-risk (RAEB-2) MDS patients (please see Table 1 A-C for patients’ characteristics). All patients were clinically refractory to Aza (Table 1A) and accordingly, primary cells from 6 patients were found to be fully resistant to Aza as shown by the lack of Aza effect on cell proliferation, (Figure 3A). Interestingly, Aca induced a dose-dependent decrease of cell proliferation in the blasts of all 6 patients that was highly significant at all the Aca concentrations used (Figure 3A). The same experiments were also conducted in primary cells from 6 AML patients clinically refractory to Aza (Table 1A-C for patients’ characteristics). Primary cells from 4 patients were totally insensitive to Aza treatment, whereas 2 patients (red symbols) were partially sensitive to the drug. Once again Aca triggered a dose-dependent and highly significant loss of cell proliferation in the 6 AML samples, independently of the level of resistance to Aza ex vivo (Figure 3B).

### 2.3. Phase I/II Clinical Trial of Aca in MDS/AML Patients

**Figure 4:**
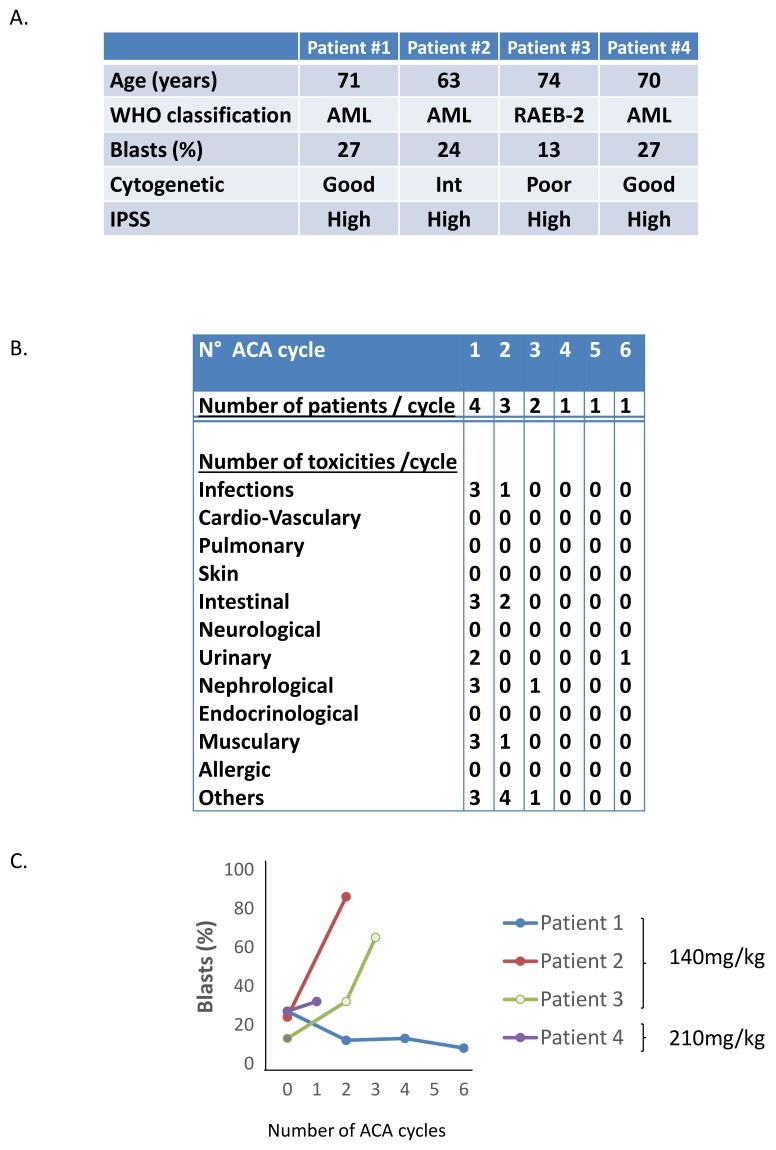
Phase I/II clinical trial of Aca in Aza-refractory MDS/AML patients. Four patients (1 MDS, and 3 AML) were enrolled in the assay and treated on Day 1 (D1), D3, D5, D8, D10 and D12 of a 28–56 day-course with Aca at 140 and 210 mg/kg. (**A**) Characteristics of the four enrolled patients. (**B**) Main side effects of the 4 enrolled patients. (**C**) Blast counts of the different patients during the course of treatment.

In a previous phase I/II clinical trial [24] in patients with relapsed/refractory chronic lymphocytic leukemia a manageable and predictable safety profile was demonstrated for Aca at single doses between 50 and 210 mg/kg. The main side effects were hyperuricemia and renal impairment that were fully reversible upon allopurinol treatment. There was evidence of anti-leukemic activity on 16/24 patients based on a 20% reduction in B-cell counts. In the same line, we initiated a dose escalation safety and tolerability effect of Aca in Aza-refractory MDS and AML patients. A total of 4 patients were included in the trial and received either 140 mg/kg (3 patients) or 210 mg/kg (1 patient) of Aca (Figure 4A). Patient #1 received 6 cycles, patient #3 received 3 cycles, patients #2 and patient #4 received 2 cycles of Aca. Two patients progressed on Aca (patients #2 an #3) as assessed by the percentage of blast count, one was stable (patient #4). However, the treatment was stopped after 2 to 3 cycles of Aca due to serious renal toxicities (Figure 4B). Patient #1, for whom 6 cycles of Aza were performed, exhibited a very strong reduction (50%) of his blast count after only 2 cycles of Aca and more than 70% after 6 cycles (Figure 4C). Despite encouraging results for one AML patient (patient #1), the trial was stopped essentially due to grade 3 urinary and renal toxicities, that were however reversible after stopping Aca (Figure 4B).

### 2.4. Medullary Stromal Cells Inhibit Aca-Induced Cell Death

**Figure 5:**
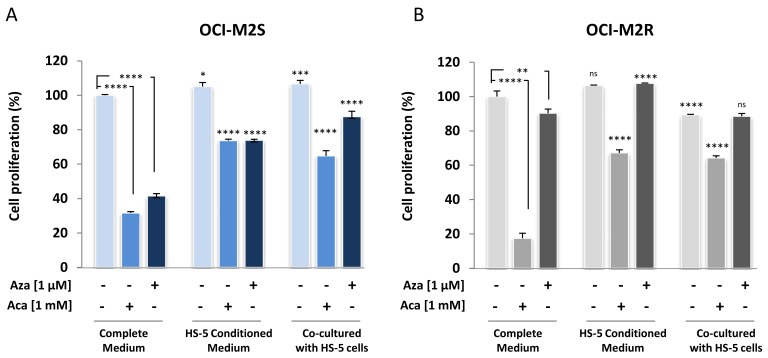
Aca fails to induce its antileukemic effect in MDS/MSC co-culture experiments. (**A**) OCI-M2S cells were growth in complete, in HS-5 conditioned medium or co-cultured with HS-5 stromal cells. Cells were then treated with 1 mM Aca or 1µM Aza. 48 h later, loss of cell proliferation was assessed using the XTT assay as described in Figure 1. Each assay was performed in quadruplicate. (**B**) OCI-M2R cells were growth in complete, in HS-5 conditioned medium or co-cultured with HS-5 cells. Cells were then treated with 1 mM Aca or 1µM Aza. 48 h later, loss of cell proliferation was assessed using the XTT assay as described in Figure 1. Each assay was performed in quadruplicate. Statistical analyzes of histograms for “HS-5 Conditioned Medium” and “Co-cultured with HS-5” conditions are compared to the “Complete Medium” used as control. * *p* < 0.05, ** *p* < 0.01, *** *p* < 0.001, **** *p* < 0.0001, ns: not significant.

We next assessed the ability of Aca and Aza to induce cell death in OCI-M2S and OCI-M2R cells co-cultured with human medullary stromal HS-5 cells or HS-5 conditioned medium. As expected, Aza efficiently killed OCI-M2S cells at 1 µM (Figure 5A) unlike resistant cells (Figure 5B). Importantly, when OCI-M2S cells were co-cultured for 48 h with stromal cells or in the presence of conditioned medium of HS-5 stromal cells, Aza was strikingly less efficient (Figure 5A). Aca killed OCI-M2S and OCI-M2R cells at 1 mM (Figure 5A,B). Co-culture conditions using either OCI-M2S (Figure 5A) or OCI-M2R (Figure 5B) and a monolayer of HS-5 stromal cells were next performed. Stromal cells diminished Aca-induced cell death in both cell lines (Figure 5A,B). We also wondered whether conditioned medium from HS-5 stromal cells could alone inhibit the effect of Aca. To this aim, OCI-M2S and OCI-M2R cells were grown 1h in complete or HS-5 conditioned medium and next treated with 1 mM Aca. 48 h later, cell proliferation was analyzed (Figure 5A,B). This last experiment clearly shows that the conditioned medium of HS-5 stromal cells inhibited Aca-induced cell death (Figure 5A,B) to roughly the same extend than HS5 stromal cells, suggesting the ability of factors present in the medium partially block antileukemic effect of Aca. In conclusion, even in the presence of HS5 stromal cells or conditioned media collected from HS5 stromal cells, Aca maintained a significant antileukemic activity on OCI-M2R cells.

## 3. Discussion

MDS defines a group of heterogenous hematologic malignancies that frequently evolved to acute myeloid leukemia. Aza, is an essential treatment for both high-risk MDS and AML patients. However, resistance to Aza is a recurrent clinical problem in treated patients and there is currently no therapeutic option for patients after Aza failure. In the present study, we demonstrate that Aca is able to bypass Aza resistance in MDS and AML cell lines and more interestingly in patient’s samples. We initiated a dose escalation safety and tolerability effect of Aca in Aza-refractory MDS and AML patients. Despite a very good response in one out 4 patients, we stopped this trial because both doses of Aca (140 and 210 mg/kg) caused serious renal side effects in several patients.

We have previously reported that Aca induces autophagic cell death in Imatinib-sensitive and resistant K562 BCR-ABL expressing CML cell lines. Aca was also very efficient to trigger tumor regression in a xenografted model of imatinib-resistant K562 cells in vivo [11]. From a mechanistic point of view, recent work from the literature also suggested that Aca eradicates CML cells by suppressing activation of the mTOR pathway in BCR-ABL expressing cells [22]. Activation of AMPK or conversely inhibition of mTOR appear therefore as pertinent therapeutic options for the treatment of BCR-ABL expressing malignancies raising the potential use of AMPK activators such as Aca or Metformin in the treatment of refractory CML and Ph(+) acute lymphoblastic leukemia [22].

Previously, we reported that Aca exerts its antileukemic effect by a caspase-independent cell death pathway in CML cells. In the present study, we extended these findings to MDS and AML cell lines and patients. In addition although the mode of cell death induced by Aca was not mechanistically identified in the present study, we established that this drug was unable to induce apoptosis in MDS and AML cell lines and exerted only a minimal effect on autophagy induction, in agreement with an unidentified cell death mechanism.

Aca, has achieved phase I/II clinical trials in B-cell chronic lymphocytic leukemia [24]. A modest reduction of B cell count (20%) was observed for two-thirds of patients. We show here that Aca has also shown some promising anti-neoplastic properties in MDS but also non-negligible side effects at high doses that preclude its use in elderly patients. Nevertheless, the present data underscoring the efficacy of Aca in vitro and in vivo and in one out 4 patients in Aza-resistant MDS/AML cell lines pave the way for reassessing the effect of low and more tolerated doses of Aca (<140 mg/kg/day) in Aza refractory MDS/AML patients. The end pathway major metabolite of Aca is uric acid. In this context, the rapid and effective management of uric acid overload at this dose could help to analyze the anti-leukemic effects of Aca in the longer term.

It is well documented that the bone marrow environment greatly impacts drug availability and efficiency. Of note, it was recently reported that MSCs could participate to the mechanisms of chemoresistance to Aza in MDS. The data presented herein show that factors secreted in the human bone marrow microenvironment by stromal cells can impair the response to Aza, but only moderately affect the response to Aca. We have recently derived new Aca analogs that are one thousand-fold more efficient than Aca [25]. The effect of these inhibitors are currently evaluated in MDS and AML cell lines.

## 4. Materials and Methods

### 4.1. Reagents and Antibodies

IMDM and fetal calf serum (FCS) were purchased from Invitrogen (Villebon sur Yvette, France). Sodium fluoride, sodium orthovanadate, phenylmethylsulfonyl fluoride (PMSF), aprotinin, leupeptin and Aza were purchased from Sigma (Saint-Louis, MO, USA). Anti-PARP, anti-LC3, anti-caspase 3 and anti-rabbit antibodies were from Cell Signaling Technology (Beverly, MA, USA). Peroxidase-conjugated anti-goat and Peroxidase-conjugated anti-mouse antibodies were from Dakopatts (Glostrup, Denmark). Anti-Actin antibodies were from Santa Cruz Biotechnology (Heidelberg, Germany).

### 4.2. Cell Lines

The human cell lines OCI-M2S and OCI-M2R cells have been generated by iterative additions of increasing concentrations of Aza starting from 0.1 μM until 10 μM. OCI-M2S cells were cultured in the same culture conditions than OCI-M2R cells, all along the selection process that lasted several months. Sensitive and resistant cells were cultured in IMDM medium complemented with 20% SVF, 50 U/mL penicillin, 50 mg/mL streptomycin, and 1 mM pyruvate under 5% CO_2_ in a humidified incubator.

### 4.3. Bone Marrow Samples

Mononuclear cells were isolated by Ficoll gradient centrifugation from bone marrow cells of MDS/AML patients and were suspended in IMDM medium containing 10% FCS, 50 U/mL penicillin, 50 mg/mL streptomycin, and 1 mM pyruvate under 5% CO_2_ in a humidified incubator. Fresh bone marrow samples were treated by Aza or indicated concentration of Aca or other drugs. All patients had diagnosis of MDS with less than 30% of blasts and IPSS scoring intermediate-2 or high or AML. The diagnosis of MDS or AML was based on standard WHO criteria [26]. Patients (Pts) were to receive Aza at the FDA/EMEA approved schedule (75 mg/m²/d, 7 d/ 4 weeks). Pts having received ≥1 cycle of Aza and who had bone marrow evaluation after ≥4 cycles, or who died or progressed before completion of 4 cycles were considered evaluable (the last 2 groups were considered as treatment failures). Responses were scored according to IWG 2006 criteria for MDS [27] and for AML [28]. All patients under treatment with Aza were included in protocol NCT01210274 (www.clinicaltrials.gov). Informed consent was obtained for all patients.

### 4.4. Assessment of Cell Proliferation:

Cell proliferation was determined with XTT assay purchased from Sigma Roche Applied Science (Penzberg, Germany). Cells (20 × 10^3^) were incubated in a 96-well plate with the indicated concentration of cell death at a final volume of 100 μL. After 24 h, 50 μL of XTT reagent (sodium 3’-[1-(phenylaminocarbonyl)-3,4-tetrazolium]-bis(4-methoxy-6-nitro)benzene sulfonic acid hydrate) was added to each well. The assay is based on the cleavage of the yellow tetrazolium salt XTT to form an orange formazan dye in metabolically active cells. The absorbance of the formazan product, reflecting cell proliferation, was measured at 490 nm. Each assay was performed in quadruplicate.

### 4.5. Western Blot Analysis

After stimulation with various effectors for 24 h, cells were harvested and lysed in buffer containing 1% Triton X-100 and supplemented with protease and phosphatase inhibitors (Roche Diagnostics). Lysates were pelleted, and 50 μg of protein were separated on 12% polyacrylamide gel and transferred onto polyvinylidene difluoride (PVDF) membrane (Immobilon-P, Millipore, Bedford, MA, USA). After blocking non-specific binding sites, the membranes were incubated with specific antibodies, washed three times and finally incubated with HRP-conjugated antibody for 1 h at room temperature. Immunoblots were revealed using the enhanced chemiluminescence detection kit (Amersham Biosciences, Uppsala, Sweden).

### 4.6. Caspase 3 Activity Assay

After stimulation, cells were lysed at 4 °C in lysis buffer (0.4 M Na Phosphate PH 6, 150 mM NaCl, 4 mM EDTA, 1 mM PMSF, 10 mg/mL aprotinin and 1% Triton X-100) and lysates were cleared at 10,000 g for 15 min at 4 °C. Each assay, in quadruplicates, was performed with 15 mg of protein prepared from control or stimulated cells. Briefly, cellular extracts were then incubated in a 96-well plate, with 0.2 mM Ac-DEVD-AMC as substrates for various times at 37 °C and Caspase 3 activity is measured after emission at 460 nm (excitation at 390 nm) with or without 1 mM Ac-DEVD-CHO. Enzyme activities were expressed in arbitrary units/mg of protein.

### 4.7. Phase I-II Clinical Trial of Aca in MDS/AML Patients

GFM Acadesine was a phase I-II trial of Acadesine in IPSS high and int 2 myelodysplastic syndromes, acute myeloid leukemia with 20–30% marrow blasts and chronic myelomonocytic leukemia type 2 patients not responding to azacitidine or decitabine for at least 6 courses or relapsing after a response. The study was sponsored by Groupe Francophone des Myélodysplasies (GFM). GFM-Acadesine received authorization from the French regulatory authority, ANSM, on 29-Mar-2013. The primary objective was to determine the maximal tolerated dose (MTD) and dose limiting toxicities (DLTs) of increasing doses of IV Acadesine administered on D1, D3, D5, D8, D10 and D12 of a 28 to 56 day-course. The secondary objectives were to evaluate the response rate (according to 2006 modified IWG criteria) and duration, hospitalization duration rates of re-hospitalization for non-hematological toxicities, severe bleeding or febrile neutropenia. EudraCT number: 2012-003120-21.

### 4.8. Statistical Analysis

All data are presented as mean ± S.D. *p*-values were determined using Prism V7.0 software (GraphPad, La Jolla, CA, USA). Unless stated otherwise in the figure legend, comparisons of different groups were made with the one-way ANOVA test with Bonferroni posttest. *p*-values <0.05 (*), <0.01 (**), <0.001 (***) and <0.0001 (****) were considered statistically significant.

## Figures and Tables

**Table 1:** Patients’ characteristics

(A)		*n* = 12	
WHO Classification			
RAEB-2		6 (50%)	
AML 20–30 %		6 (50%)	
**IPSS Cytogenetic Risk**			
Good		3 (25%)	
Intermediate		3 (25%)	
Poor		6 (50%)	
**Cytogenetics Abnormalities**			
Normal Karyotype		3 (25%)	
+8		1 (8%)	
7 Abnormalities		3 (25%)	
5 Abnormalities		3 (25%)	
Complex > 3 Abnormalities		2 (17%)	
**IPSS Classification**			
Low		0	
Intermediate 1		0	
Intermediate 2		2 (17%)	
High		10 (83%)	
**Aza Response Status**			
Failure		8 (67%)	
Relapse		4 (33%)	
**Number of Aza Cycles before Relapse or Failure**		6 [1,2,3,4,5,6,7,8,9,10,11,12,13,14,15,16,17,18,19,20,21,22,23,24,25,26,27]	
**(B)**			
**Patients**	**Disease**	**IPSS Cytogenetic Risk**	**Cytogenetic Abnormalities**
**#1**	RAEB-2	Intermediate	Normal
**#2**	RAEB-2	Intermediate	Normal
**#3**	RAEB-2	Intermediate	Normal
**#4**	RAEB-2	Intermediate	47,XY,+8
**#5**	RAEB-2	Intermediate	Normal
**#6**	RAEB-2	Intermediate	46,XY(del(9)(q12q31)/47,idem,+21
**#7**	AML	Poor	45,XX,-7
**#8**	AML	Poor	45,XX,t(3;11)(q22,q33),del(9)(q22,q33),-7
**#9**	AML	Poor	45,XX,-7, inv(3), del11q
**#10**	AML	Intermediate	46,XY,inv(3)(q21q26)
**#11**	AML	Poor	46,XX,del(5)(q13q33)/47, idem, del(1)(p3?4),+mar/46,XX
**#12**	AML	Good	45,X,-Y
**(C)**			
**Patients**	**IPSS Classification**	**AZA Response Status**	**Number of AZA Cycles before Relapse or Failure**
**#1**	High	Failure	3
**#2**	Intermediate 2	Relapse	27
**#3**	High	Failure	6
**#4**	High	Relapse	9
**#5**	Intermediate 2	Relapse	19
**#6**	High	Relapse	26
**#7**	High	Failure	6
**#8**	High	Failure	4
**#9**	High	Failure	6
**#10**	High	Failure	1
**#11**	High	Failure	6
**#12**	High	Failure	6
